# An observational study of alemtuzumab following fingolimod for multiple sclerosis

**DOI:** 10.1212/NXI.0000000000000320

**Published:** 2017-01-10

**Authors:** Mark Willis, Owen Pearson, Zsolt Illes, Tobias Sejbaek, Christian Nielsen, Martin Duddy, Kate Petheram, Caspar van Munster, Joep Killestein, Clas Malmeström, Emma Tallantyre, Neil Robertson

**Affiliations:** From the Department of Neurology (M.W., E.T., N.R.), Institute of Psychological Medicine and Clinical Neuroscience, Cardiff University, University Hospital of Wales; Department of Neurology (O.P.), Morriston Hospital, Heol Maes Eglwys, Morriston, Swansea, UK; Departments of Neurology (Z.I., T.S.) and Clinical Immunology (C.N.), Odense University Hospital, University of Southern Denmark; Department of Neurology (M.D.), The Royal Victoria Infirmary, Newcastle upon Tyne; Department of Neurology (K.P.), Sunderland Royal Hospital, UK; VU University Medical Center (C.v.M., J.K.), Amsterdam, the Netherlands; and Department of Neurology (C.M.), Sahlgrenska Academy at the University of Gothenburg, Institute of Clinical Neuroscience and Physiology, Gothenburg, Sweden.

## Abstract

**Objective:**

To describe a series of patients with relapsing multiple sclerosis (MS) who experienced significant and unexpected disease activity within the first 12 months after switching from fingolimod to alemtuzumab.

**Methods:**

Patients with relapsing MS treated sequentially with fingolimod then alemtuzumab who experienced significant subsequent disease activity were identified by personal communication with 6 different European neuroscience centers.

**Results:**

Nine patients were identified. Median disease duration to alemtuzumab treatment was 94 (39–215) months and follow-up from time of first alemtuzumab cycle 20 (14–21) months. Following first alemtuzumab infusion cycle, 8 patients were identified by at least 1 clinical relapse and radiologic disease activity and 1 by significant radiologic disease activity alone.

**Conclusions:**

We acknowledge the potential for ascertainment bias; however, these cases may illustrate an important cause of reduced efficacy of alemtuzumab in a vulnerable group of patients with MS most in need of disease control. We suggest that significant and unexpected subsequent disease activity after alemtuzumab induction results from prolonged sequestration of autoreactive lymphocytes following fingolimod withdrawal, allowing these cells to be concealed from the usual biological effect of alemtuzumab. Subsequent lymphocyte egress then provokes disease reactivation. Further animal studies and clinical trials are required to confirm these phenomena and in the meantime careful consideration should be given to mode of action of individual therapies and sequential treatment effects in MS when designing personalized treatment regimens.

The pathophysiology of multiple sclerosis (MS) involves a complex interplay of immunologic factors, including a pivotal role for T and B lymphocytes. As a result, current disease-modifying treatments (DMTs) are targeted at immune cell trafficking, lymphocyte function, and lymphodepletion.^1^ With a growing range of interventions now available for relapsing disease, it has become of increasing importance to understand their place and timing in individualized therapy. In addition, the immediate and long-term consequences of sequential drug use and the optimum order in which they should be used is unclear but may significantly affect efficacy, adverse events, and longer-term immunocompetence. While awaiting clinical studies to address these issues, observations from clinical practice will be of value in guiding current protocols, generating hypotheses, and informing trial design.

We report a case series of 9 patients from 6 European neuroscience centers who switched to alemtuzumab following incomplete suppression of inflammatory disease activity by fingolimod. Despite the established efficacy of alemtuzumab, which reduces relapse frequency by up to 74% against an active comparator (interferon [IFN]–β-1a), and annualized relapse rates (ARR) from 2.1 to 0.2 in open-label studies,^1^ all patients experienced a lack of response to alemtuzumab with significant new disease activity. We hypothesize that this occurs as a result of prolonged lymph node sequestration of circulating lymphocytes following fingolimod withdrawal, which in turn limits the efficacy of alemtuzumab. In humanized mice, CD52-positive lymphocyte lysis is more profound in the peripheral circulation compared to the degree of depletion in lymphoid organs^2^ and suggests that in some patients lymphocytes may remain concealed from the usual therapeutic effects of alemtuzumab. This may have important implications for sequential drug selection and washout periods in a subset of patients who switch from fingolimod or drugs with similar biological mechanisms.

## METHODS

Patients with relapsing MS treated sequentially with fingolimod followed by alemtuzumab were identified as having significant and unexpected subsequent disease activity by personal communication with 6 European neuroscience centers. History was obtained by clinical note review and radiologic outcomes analyzed.

## RESULTS

Table 1 summarizes clinical information for each patient. All values are median times with ranges in parentheses. Age at disease onset was 22 (8–32) years and disease duration to alemtuzumab treatment 94 (39–215) months. Follow-up from first alemtuzumab cycle was 20 (14–21) months. Time on fingolimod was 13 months (5–33). All patients had received additional DMTs prior to fingolimod: 7 received IFN-β and 2 natalizumab as first-line therapy. Of patients commenced on IFN, 6 escalated to natalizumab, with 1 patient proceeding directly to fingolimod (table 2). Clinical disease or radiologic disease activity were principal reasons for switching from IFN-β and glatiramer acetate but in 8 patients who transitioned from natalizumab to fingolimod only 1 switched as a result of clinical disease activity.

**Table 1:**
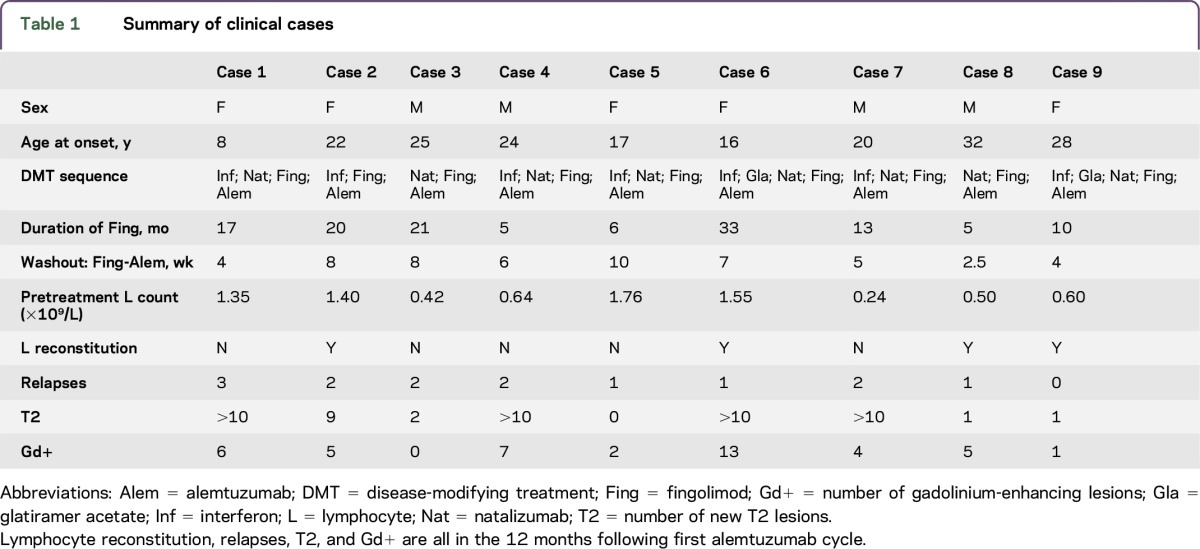
Summary of clinical cases

**Table 2:**
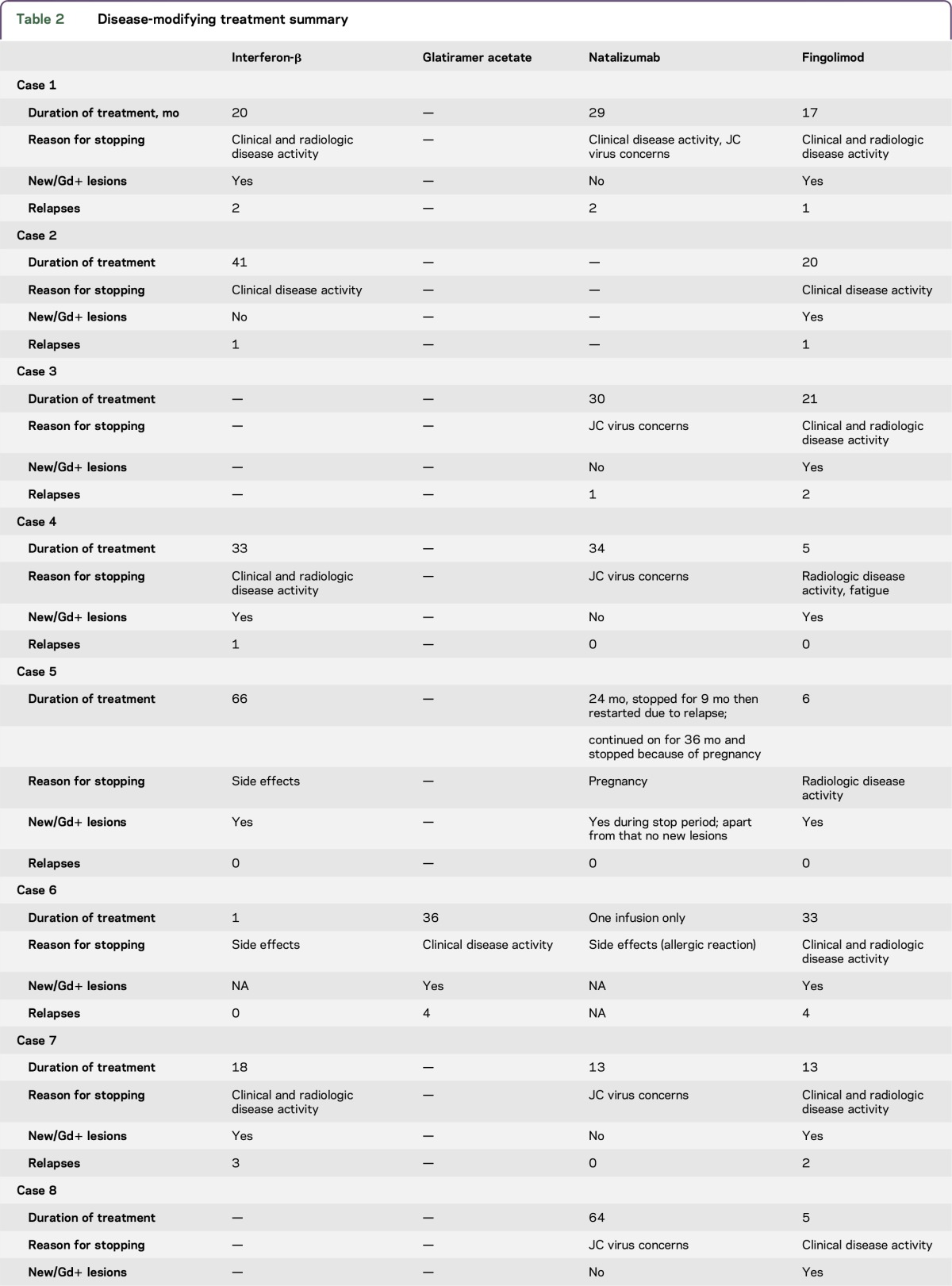

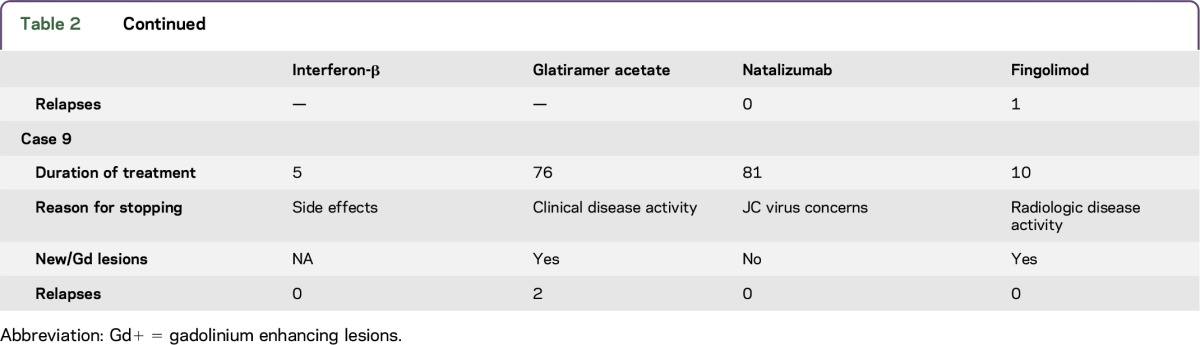
Disease-modifying treatment summary

Median fingolimod washout period was 6 (4–10) weeks prior to first alemtuzumab cycle. At first treatment, 5 patients had lymphocyte counts below normal range (median 0.64 × 10^9^/L, range 0.24–1.76 × 10^9^) and in 4 patients lymphocytes reconstituted more rapidly than expected (median 3 months). Following initial infusion, 8/9 patients experienced at least 1 clinical relapse in the first 12 months (ARR 1.6) with all patients having radiologic evidence for new disease activity on MRI in the form of new T2 lesions or gadolinium (Gd)–enhancing lesions (table 1 and figure). Median time to relapse following alemtuzumab induction was 4.5 months.

**Figure:**
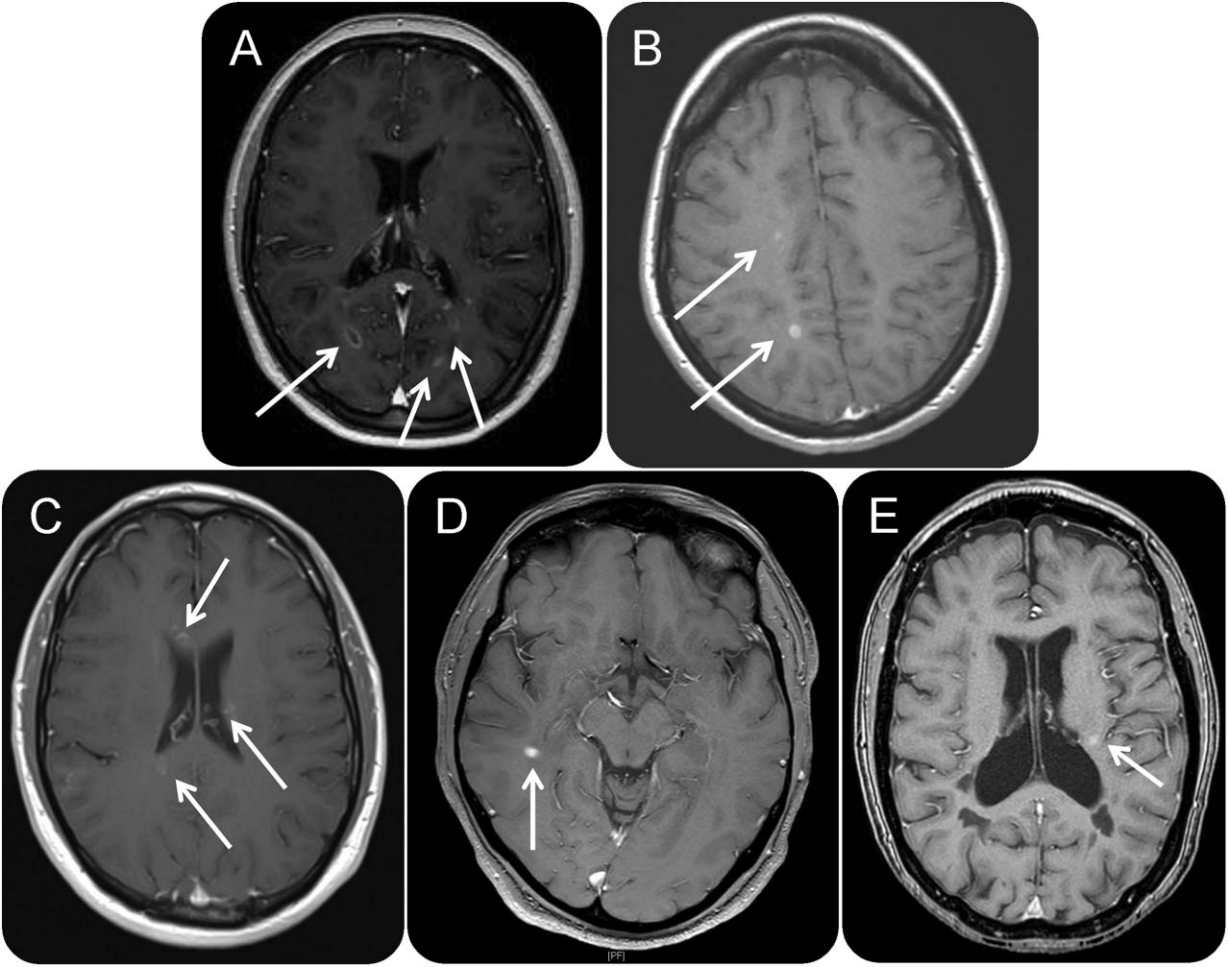
Axial T1 plus contrast images (time post alemtuzumab) (A) Case 1 (8 months). (B) Case 2 (10 months). (C) Case 4 (8 months). (D) Case 8 (7 months). (E) Case 9 (6 months). White arrows = new gadolinium-enhancing lesions.

Despite the presence of disease activity in the first 12 months, all patients went on to receive the second planned infusion of alemtuzumab and further follow-up is currently available for a mean of 6 months from second treatment cycle. During this period, 8 patients have been relapse-free (combined ARR 0.3). Seven patients have had further imaging: 4 patients were radiologically stable and 3 developed new T2 lesions, 1 of whom had a single new Gd-enhancing lesion. Two patients have not yet undergone further interval imaging.

## DISCUSSION

Fingolimod is an oral DMT causing internalization of S1P type 1 receptors and inhibition of lymphocyte trafficking from secondary lymphoid organs into the peripheral circulation. This results in a reduction of the number of lymphocytes available to cross the blood–brain barrier into the CNS, which is thought to be responsible for reducing disease activity.^3^

Alemtuzumab is a humanized monoclonal antibody targeted against the cell surface protein CD52. Treatment results in a rapid and profound reduction in peripheral lymphocytes as a result of antibody-dependent cell-mediated cytotoxicity, complement-dependent cytolysis, and induction of apoptosis.^1^ Repopulation occurs through proliferation of mature lymphocytes that escape deletion or by thymic repopulation from CD52-negative hematopoietic precursors.^4^ A relative increase in regulatory CD4^+^ T cells (Tregs) is observed following treatment and repopulation^5^ together with an increased representation of memory T lymphocytes.^6^ This pattern of subsequent reconstitution of the immune system is thought to be beneficial for MS.

Fingolimod has a pharmacologic half-life of 6–9 days^7^ and lymphocytes would be expected to normalize 2–4 weeks after discontinuation.^3^ However, there are case reports of prolonged lymphopenia following prolonged drug exposure, up to 37 months after discontinuation.^8^ Following discontinuation, there is also a risk of rebound disease activity 2–4 months from the time of withdrawal.^3^ In animal models, rebound is preceded by a burst of S1P1 overexpression in trapped lymphocytes, which also correlates with subsequent massive lymphocyte egress and CNS infiltration. In addition, Treg functionality is impaired following cessation of fingolimod, which may also contribute to rebound activity.^3^ It has therefore been suggested that patients continue on an alternative DMT after fingolimod discontinuation, preferably until peripheral lymphocyte counts have normalized. There is currently no consensus as to which subsequent therapeutic agent is optimal.

Following alemtuzumab, there is a significant reduction in relapse frequency, with phase III trials demonstrating 60%–74% of patients at 24 months having freedom from clinical disease, defined as the absence both of relapses and sustained accumulation of disability. In addition, only 7%–9% of patients had gadolinium-enhancing lesions at 24 months on annually performed MRI.^1^

In our own published cohort^9^ of 100 patients treated with alemtuzumab, only 4 patients experienced >1 relapse in the 12 months after initial infusion. In contrast, all 9 patients reported here experienced significant and unexpected disease activity. We consider the radiologic findings of these cases particularly striking, especially given the relatively short follow-up time.

We hypothesize that the significant and unexpected disease activity in these cases is caused by sequestrated lymphocytes, which remain concealed from the usual therapeutic effects of alemtuzumab, which has a relatively short half-life of between 7 and 21 days.^10^ Following this period, surviving CD52-positive lymphocytes then egress from lymph nodes, initiating the observed inflammatory disease activity. Variability in lymphocyte reconstitution may also contribute to these effects: in case 2, it took only 2 months for the peripheral lymphocyte count to return to normal range following the first cycle of alemtuzumab and 1 month after the second cycle. This is considerably faster than the reported mean of 7.1 months for B cells and 20 and 35 months for CD8^+^ and CD4^+^ T cells, respectively.^1^

Other factors that may influence disease activity include S1P1 overexpression and subsequent lymphocyte egress contributing to accelerated repopulation^3^ and modification of the circulating immune repertoire by fingolimod altering the expected deletion pattern following alemtuzumab. In support of this, 5 patients remained lymphopenic at the start of treatment. In the 4 who had normal lymphocyte counts, it could be speculated that an altered repopulating immune repertoire and impaired Treg functionality may be contributing to the observed disease activity. Alternatively, it may be that for some patients, pathogenic lymphocytes may continue to be released from the lymphoid system over a more prolonged period. While on fingolimod, the mean ARR was 1, increasing to 1.6 in the 12 months following alemtuzumab then reducing to 0.3 following the second dose to date, lending some support to the hypothesis that, after a period, sequestrated lymphocytes eventually become available for depletion by alemtuzumab.

Across the 6 centers, a total of 174 patients have been treated with alemtuzumab. Of these, 36 received fingolimod prior to administration of alemtuzumab. Therefore, these 9 patients with unexpected and significant disease activity following alemtuzumab represent 25% of the fingolimod-alemtuzumab cohort. Detailed outcome data are not currently available on all 174 patients treated with alemtuzumab but would clearly represent a valuable resource for more detailed investigation. Therefore, although we recognize the potential for selection bias in this case series, the degree of disease activity observed in these cases spontaneously and independently caught the attention of clinicians in different centers and may represent an important cause of lack of therapeutic efficacy in a group of patients most at need of disease control. Further studies including detailed immunophenotyping are required to confirm these phenomena and may have relevance in understanding causes of disease reactivation in general. In addition, future trials addressing longer-term outcomes of induction and escalation paradigms need to be designed in order to guide clinicians on strategies for treatment switches. In particular, careful consideration needs to be given to mode of action of individual therapies and sequential treatment effects.

## GLOSSARY

ARR: annualized relapse rate
DMT: disease-modifying treatment
IFN: interferon
MS: multiple sclerosis
Treg: regulatory CD4^+^ T cell
